# Promoting equitable global health research: a policy analysis of the Canadian funding landscape

**DOI:** 10.1186/s12961-017-0236-2

**Published:** 2017-08-29

**Authors:** Katrina Plamondon, Dylan Walters, Sandy Campbell, Jennifer Hatfield

**Affiliations:** 1 Research Department, Interior Health, Kelowna, BC Canada; 2 School of Nursing, University of British Columbia, 3333 University Drive, Kelowna, BC V1V 1V7 Canada; 3 Canadian Centre for Health Economics, Institute of Health Policy, Management and Evaluation, University of Toronto, 155 College Street, 4th Floor, Toronto, ON M5T 3M6 Canada; 4 Independent consultant, Taos, New Mexico USA; 5 Global Health & International Partnerships Office, Cumming School of Medicine, University of Calgary, Dean’s Office, 7th Floor, Teaching Research & Wellness Building, 3330 Hospital Drive NW, Calgary, AB T2N 4N1 Canada

**Keywords:** Global health research, Funding, Research policy, Canada

## Abstract

**Background:**

Recognising radical shifts in the global health research (GHR) environment, participants in a 2013 deliberative dialogue called for careful consideration of equity-centred principles that should inform Canadian funding polices. This study examined the existing funding structures and policies of Canadian and international funders to inform the future design of a responsive GHR funding landscape.

**Methods:**

We used a three-pronged analytical framework to review the ideas, interests and institutions implicated in publically accessible documents relevant to GHR funding. These data included published literature and organisational documents (e.g. strategic plans, progress reports, granting policies) from Canadian and other comparator funders. We then used a deliberative approach to develop recommendations with the research team, advisors, industry informants and low- and middle-income country (LMIC) partners.

**Results:**

In Canada, major GHR funders invest an estimated CA$90 M per annum; however, the post-2008 re-organization of funding structures and policies resulted in an uncoordinated and inefficient Canadian strategy. Australia, Denmark, the European Union, Norway, Sweden, the United Kingdom and the United States of America invest proportionately more in GHR than Canada. Each of these countries has a national strategic plan for global health, some of which have dedicated benchmarks for GHR funding and policy to allow funds to be held by partners outside of Canada. Key constraints to equitable GHR funding included (1) funding policies that restrict financial and cost burden aspects of partnering for GHR in LMICs; and (2) challenges associated with the development of effective governance mechanisms. There were, however, some Canadian innovations in funding research that demonstrated both unconventional and equitable approaches to supporting GHR in Canada and abroad. Among the most promising were found in the International Development Research Centre and the (no longer active) Global Health Research Initiative.

**Conclusion:**

Promoting equitable GHR funding policies and practices in Canada requires cooperation and actions by multiple stakeholders, including government, funding agencies, academic institutions and researchers. Greater cooperation and collaboration among these stakeholders in the context of recent political shifts present important opportunities for advancing funding policies that enable and encourage more equitable investments in GHR.

## Background

Global health research (GHR) prioritises health equity and improved well-being for all people worldwide. It involves transnational health issues, determinants and solutions, involves collaboration across many disciplines within and beyond the health sciences, and is undertaken in order to inform (and be informed by) policy at the local, national and global level [1–3]. Given the central value and focus of GHR on health equity, equity in research-related practices and policies are important foundations for this field of research. Between 2013 and 2015, people involved in doing, teaching about, supporting and using GHR in Canada contributed to a series of dialogue-based studies aimed at articulating a shared vision for action. Among the outputs of this work are the study reported here and the creation of a set of equity-centred principles for GHR [4]. These six equity-centred principles set an aspirational standard for ethical, equitable engagement in GHR, including investments and supports for GHR through funding policies. These principles, and the shared concerns of participants in the Gathering Perspectives Studies, served as the foundation for the policy analysis presented here.

Canadian investments in GHR have, like in other fields of health research, been subject to a dynamic policy environment over the last decade. Following the 2008 economic recession, there was an unprecedented reorganisation of the GHR funding landscape in Canada. As new global public health threats emerged, major funding bodies underwent reform and private investors expanded their involvement in research and development. These shifts occurred amid intensified pressure to demonstrate results and value for money. While the explicit rationale for the structural changes in Canada’s GHR funding landscape was to strengthen its position as a world leader in research, the underlying reasons for changes are difficult to discern and their future impacts unknown.

In 2011, the Canadian Academy of Health Sciences called upon Canadians to play a more strategic role in global health [5]. The Canadian Coalition for Global Health Research (CCGHR), a network of people interested in promoting better and more equitable health worldwide through the production and use of knowledge, responded by leading research^1^ that invited actors in the GHR community to engage in dialogue on the state of health research and practice in Canada. Participants in this research identified a need for tools that could support navigation of a changing funding landscape and inform the evolution of policies and practices. The purpose of this study was to examine the GHR funding system in Canada and comparator countries to better understand the current funding landscape and identify promising practices that could inform equitable approaches to GHR funding. The recommendations stemming from this analysis may inform a dialogue on Canada’s strategic role in enabling equitable and ethical GHR [5, 6].

## Methods

This study involved analysing funding policies for their alignment with equity-centred GHR, using the CCGHR Principles for GHR [4] as an analytical tool to guide assessment of equitable and ethical GHR policies and practices. For the purposes of the study, we defined policy as anything that explicitly or implicitly determined the ways in which GHR grants could be prepared, used or administered, as well as guidelines, statements or direct policies that delineate funding bodies’ investments in GHR. This includes funding practices that may not be documented as formal policies, but that constitute a routine or typical way a funding body engages with GHR (e.g. funding agencies’ practices in selecting reviewers or monitoring competition outcomes for bias or compliance with GHR eligibility policies; university norms for administration of grant monies).

Grounded in a reflexive approach [7, 8], this study centred around three analytical questions – (1) how is GHR conceptualised in funding policies; (2) how are equity-centred principles of GHR reflected (or not) in policies (Table 1); and (3) how are the interests of intended beneficiaries considered (or not) in these policies. The approach to policy analysis was guided by the Ideas, Interests and Institutions conceptual framework [9].

**Table 1:** Criteria for assessing equity in funding policies

CCGHR Principles for Global Health Research	Description	Potential applications in funding policy
Authentic partnering	Building equity and reciprocity considerations into research partnerships, including the ways in which research partnerships enable fair distribution of resources, power and benefits	• Attention to research teams’ partnership structures, distribution of resources, degree of participation and/or collaboration (e.g. through team composition, budget) • Requiring transparency in intention to adopt equitable, ethical partnering strategies • Setting expectations for GHR to recognise and mitigate power imbalances (e.g. between Canadian researchers and their LMIC partners) • Requiring the use of partnership assessment tools or process evaluation, including research on the use of these tools
Inclusion	Intentionally providing people who have been historically marginalised opportunities to engage in research processes	• Promoting integrated knowledge translation or engaged study designs that include research users in identifying and defining research problems, setting priorities, articulating questions, conducting research and designing dissemination products • Setting budget guidelines for inclusion of trainees or mentees (e.g. emerging leaders), particularly from partner countries
Shared benefits	Being attentive to and mitigating the potential for research to benefit the principal investigator more than the communities or partners with whom they are working	• Setting expectations about research outputs that include benefits beyond traditional academic outputs (i.e. publications) • Requiring documentation of how research teams are attempting to achieve reciprocity • Encouraging budget allocation that prioritises equitable resourcing for LMIC partners to benefit as trainees and/or attend conferences • Encouraging budget allocation to post-product/post-trial benefits for communities involved in randomised controlled trials • Assessing for equity intentions in access to evidence, including open access policies for publications and in data repositories
Commitment to the future	Honouring global citizenship and humanity’s shared future in the world, including prioritising research that contributes to a better, more equitable world for future generations	• Examining how a particular project fits within a broader relationship or programme of research • Providing funding for multi-year projects • Inviting research specific to global sustainability and inherently global health issues such as climate change or globalisation • Assessing grants for alignment with human rights language and/or work • Encouraging budget allocated to trainees and mentorship • Funding multi-institution teams or networks • Investing in harmonisation efforts
Responsiveness to causes of inequities	Recognising, examining and interrupting root causes of health inequities through research	• Ensuring reviewers are familiar with the evidence about root causes of health inequities • Assessing grants for efforts to recognise, examine and interrupt root causes of health inequities • Encouraging applied and/or interventional research that aims to recognise, examine or interrupt root causes of health inequities • Encouraging research on research to illuminate and interrupt inequitable research practices or study designs
Humility	Positioning researchers in a position of learning, rather than knowing	• Encouraging adaptive, responsive or supportive steps for investing in research and/or knowledge translation (e.g. formative evaluations that open possibilities for adjusting plans) • Inviting integrated knowledge translation, action research, applied or engaged study designs

Two distinct datasets were used for this analysis. The first dataset was generated at a 2013 CCGHR deliberative dialogue involving participants who self-identified as having some involvement in GHR. Perspectives from within the GHR community that were reflected included those from non-governmental organisations, university administration, researchers, teachers, students, funders and private organisations involved in GHR. Participants at this event voiced concerns about issues of stability, ethics and equity for GHR funding and called on the CCGHR to undertake further policy research. The data included specific reflections on formal and informal funding policies in Canada. This dataset was re-analysed in this study for content pertaining to this study’s research questions. The HealthBridge Research Ethics Board reviewed and approved the ethics application for the study in which this deliberative dialogue was held (Certificate Number: HBREB/2013_1). Participants’ consent included acknowledgment of the possibility that their data may be used for future studies.

The second dataset was composed of literature and documents relating to selected national funders of GHR in Canada and major global comparators. Inclusion criteria for funders involved being an agency with explicit or implicit investments in GHR, degree of influence over strategy, and researcher accessibility to documents in English. We selected a heterogeneous set of funding organisations, including overseas development agencies, health research councils, development research centres and philanthropic foundations. We searched for documents that described these organisation’s values, strategies, programmes and granting policies. Documents retrieved were funding bodies’ strategic plans, progress reports and grant management policies relevant to GHR, although these varied widely in terms of scope, timeframe and level of detail. In addition, we searched academic databases, including Medline, PubMed, Scopus and Google Scholar, for peer-reviewed research on this topic. Key medical subject headings and search terms included ‘global health research’, ‘funding’ and ‘development assistance’.

Using a content analysis approach [10], and guided by the Ideas, Interests and Institutions framework, data from both datasets were coded for the forces, facilitators, barriers and gaps that shaped the structures, strategies, priorities and policies of the GHR funding landscape in Canada and abroad. Documents were coded with assistance from NVivo 10 [11]. Our analysis continued iteratively with initial findings evolving to inform coding structures and shaping new questions. The results of both the document analysis and secondary analysis of data from the deliberative dialogue were synthesised. We then used a deliberative approach [12, 13] to review the results of our analysis, debate about implications and collectively arrive at a series of recommendations. For this deliberative approach, we presented participants with a summary of our findings and asked them (1) how the results did or did not resonate with their experience; (2) to identify any gaps in our analysis; and (3) for their reactions to the series of recommendations. Participants in these deliberations represented a diverse range of perspectives in terms of their involvement in GHR, including people currently or previously involved in academic administration, government agencies, funding agencies, professorial positions, non-governmental organisations and philanthropic organisations. Perspectives from Canada, the United States of America, Europe, Africa and Central/South Asia were reflected among these contributors. Participants included the CCGHR Gathering Perspectives Study (Phase 2) research team (n = 17) as well as expert informants and stakeholders (n = 5) from Canada, comparator funding countries, and non-Canadian and international research partners representing a broad range of disciplines. In addition, deliberations included the CCGHR board (n = 11) and its University Advisory Council (composed of representatives from 23 universities across Canada). Responses to the questions we posed were provided in both verbal and written format, and were used to strengthen our analysis and refine recommendations.

## Results and Discussion

This section summarises the key findings from our analysis and provides a snapshot of the funding landscape in Canada and comparison countries.

### Global context for GHR

The global context of investments in GHR is marked by intense diversity in direction, intent and funding structures. Funders and other GHR stakeholders, both in Canada and abroad, use a varied terminology to describe the activities aligning with our definition of GHR. This may be attributable to agencies’ particular objectives and intended beneficiaries of GHR investments, which also vary widely. The stated rationale for GHR investments ranged across a wide spectrum, from the pursuit of commercialisation opportunities, through the advancement of basic and applied sciences, to eliminating the burden of diseases and poverty or supporting health as a human right. Similarly, we found that funding originating in wealthy countries flowed to a variety of targeted beneficiaries, including local researchers, institutions and populations, as well as marginalised, indigenous or resource-poor populations globally. This variability is observable among agencies that directly support GHR (e.g. funding agencies) and within general investments in global health through development assistance or other programmes that indirectly support GHR (e.g. through investment in evaluation, knowledge translation or innovation). For example, a major determinant of GHR funding among Organisation for Economic Co-operation and Development (OECD) countries is the direction and amount of foreign aid for health sector activities, which is otherwise known as development assistance for health (DAH). DAH globally climbed to a new high of US$31.3 billion in 2013, although the 3.9% growth from 2012 to 2013 falls short of the average 10% annual increases seen over the 2001–2010 period [14]. Correspondingly, there was an 18.4% average annual increase in funding for ‘global’ activities, which are defined as health research or the creation of public goods benefiting multiple regions or the whole world. Canada’s funding for DAH also had a remarkable average annual growth rate of 22.3% between 2000 and 2011 [14]. The variability in this same period among OECD countries is notable, however, ranging from 2.3% annual growth rate by France to 17.6% by the United States, which is the highest contributor in absolute terms. While there may be justification for continued growth based on need and long-term cost-benefit, there is likely more GHR funding available around the world at the present time than ever before.

This rise of GHR funding may be attributed to increased public and political awareness of global vulnerability to infectious diseases, heightened by the onset of the HIV/AIDS pandemic in the 1980s and 90s. The United Nations’ Millennium Development Goals focused political attention on the inequities between countries and championed channelling investments into priorities such as HIV/AIDS, malaria and then maternal, neonatal and child health (MNCH) [15–19]. Funding for DAH also surged as public institutions, non-profit organisations and for-profit companies became interested in undertaking GHR. This interest led to the establishment of the President’s Emergency Plan for AIDS Relief, the Global Fund for AIDS, Tuberculosis and Malaria, and the Bill and Melinda Gates Foundation (BMGF) [15, 20]. West Africa’s 2014–2015 Ebola epidemic eventually captivated the attention of global media, public attention and donors, though largely borne out of fear of cross-continental spread [21, 22], which may have overshadowed critical messages about the need to invest in strengthening health systems, the health workforce and other fundamental areas of human development in resource-poor countries to prevent epidemics.

### Canadian funders

Canadian researchers have a long history of involvement in global health initiatives, and are recognised for leadership in GHR and practice. Innovations in funding for GHR emerged through the 2001 establishment of the Global Health Research Initiative (GHRI), with their flagship Teasdale-Corti grants, which infused more than CA$18 million into GHR funding between 2003 and 2005 [23]. Growth in academic programmes focusing on global (sometimes named international) health has boomed over the last decade [24, 25]. This growing interest and investment in GHR came at a time when heightened awareness of the inherently global nature of infectious diseases was amplified by the 2004 SARS crisis [26]. Political and economic factors may have further influenced the direction of research funding agencies. The federal governments’ leadership between 2000 and 2015, however, was characterised by a series of short-term minority governments (Liberal then Conservative) that resulted in a high turnover of ministers responsible for international development and short-lived strategies, citing unprecedented economic uncertainty as a rationale for budget cuts or reorganisation [18, 27].

Based on open data sources, the primary GHR funders based in Canada at present are Grand Challenges Canada (GCC), the International Development Research Centre (IDRC), and the Canadian Institutes of Health Research (CIHR) (see Table 2 for estimated annual funding amounts). It is assumed that Global Affairs Canada (or formerly CIDA, and then DFATD) is also a major funder of GHR activities that feature under the umbrella of evaluation or innovation, but expenditure data at this level of detail was not openly available at the time of our analysis.

**Table 2:** Overview of primary Canadian funders and their estimated annual investments in global health research

		Reported global health research funding (in millions, CAD)				
Agency	Data availability	2011–12	2012–13	2013–14	2014–15	2015–16
IDRC	Spending and results for individual grants	18.663	16.822	16.73	^a^	^b^
GCC	Basic information on recipients of grants	13.85	51.83	54.5	50.54	46.34
CIHR	Overviews of individual grants and spending across grants programmes	27	31	31	30	29
	Total national estimate	59.51	99.65	102.23	80.54	75.34

^a^In 2014, IDRC removed the ‘global health policy’ category of research spending

^b^In 2015, IDRC removed the ‘global health policy’ programme area from total expenditures

*CAD* Canadian Dollar, *CIHR* Canadian Institutes of Health Research, *GCC* Grand Challenges Canada, *IDRC* International Development Research Centre

### Global Affairs Canada (formerly CIDA, then DFATD)

Foreign policy directions and aid budgets both influence the nature of evaluation and research in global health. Between 2005 and 2015, the federal government overhauled the research and development funding landscape and established new funding priorities in line with the post-recession foreign aid policy directions of the 2009 Economic Action Plan [28]. First, the Aid Effectiveness Agenda, which claimed to have untied all food aid, was implemented with a focus on 25 priority countries that would receive 90% of aid funding [29]. Then, in general, most of Canada’s DAH followed the foreign policy objectives in fragile and conflict-affected states (e.g. Afghanistan and South Sudan) and certain humanitarian crises [30]. During this time, new and targeted development priorities were selected, such as increasing food security, securing the future of children and youth, stimulating sustainable economic growth, advancing democracy, and promoting stability and security [31, 32].

Seizing the opportunity provided by hosting the 36th G8 meeting in 2010, Prime Minister Stephen Harper launched 2010’s Muskoka Initiative and its keystone commitment of CA$5 B over 5 years by member nations towards improving global MNCH. Again, in 2014, Canada convened world leaders, development financiers and global health experts at a summit to witness a pre-election re-commitment to MNCH, pledging CA$3.5 B of purportedly ‘new money’ over the next 5 years [30]. The long-term commitment to MNCH achieved through the 2010 G8 summit was recognised as both an important contribution to meeting “*badly lagging Millennium Development Goals 4 and 5*” ([33], p. 186), and a significant global governance accomplishment. As a leading national policy initiative, it both directly and indirectly influenced research agendas of both funders and researchers by elevating the perceived importance of investment in MNCH. Further, the Muskoka Initiative has merit in its clear focus, sizable evidence for the return on investment from investing in MNCH as a whole, and the promotion of evidence-informed cost-effective interventions; however, some critiques point to the less-than-subtle political motives that may have driven the investments in MNCH.

The policies of the Muskoka Initiative have been criticised as veiled attempts to garner public support by appealing to Canadians’ valuing of universal healthcare into foreign policy prior to federal elections while failing to address the social determinants of health [34], particularly that of poverty as an underlying cause of maternal and child mortality [35]. Globally, the initiative has been subject to scrutiny for paternalistic and neo-colonial policies that are “*highly problematic from a gender and development perspective*” ([35], p. 75). An examination of OECD’s open data on foreign aid spending reveals a silent budget cut – the percentage of Canada’s official development assistance (ODA) to gross national income (GNI) dropped from 0.32% in 2012 to 0.27% in 2013 [36], and is far below the internationally accepted standard of 0.7% of GNP suggested by former Canadian Prime Minister Lester Pearson in 1970. Further, Canada’s DAH dropped from CA$542 M to CA$491 M in 2012–2013 [14]. Canada now ranks fifteenth among donor nations for ODA/GNI ratio, which is hardly consistent with the government’s projected image of leadership in the field [36].

The government reportedly allowed nearly CA$800 M, or 11%, of the foreign aid budget to expire at the end of the 2013 fiscal year [37]. Unfortunately, the publicly disclosed financial reports and budget plans for DFATD do not contain a year-over-year comparison of expenditures for the transition period from CIDA in 2012–2013 to DFATD 2013–2014. Analysis of DFATD’s budget projections showed that, after the MNCH funding announcements, the annual budget for international poverty reduction remained steady at roughly CA$3.0 B [30, 38]. If the 2014 CA$3.5 B MNCH commitment is not actually ‘new money’ relative to historical levels but compared to the 2012–2013 level after the budget cut, Canada will be set up to underachieve on its commitments proclaimed at the MNCH Summit [39, 40].

With a new federal government in power, and a fresh rebranding of DFATD as Global Affairs Canada, there are numerous opportunities for the new government to support a more GHR-friendly environment in Canada. Commendably, Global Affairs Canada has already made commitments to raise the limits to percentage of grant funds that can be used for monitoring and evaluation. In June 2017, Global Affairs Canada launched a ‘feminist international assistance’ policy that prioritises a human rights approach and is accompanied by CA$150 M over 5 years [41]. The current government’s interest in using evidence to inform policy is also encouraging, as are their efforts to engage Canadians in open consultations that have key relevance for global health and GHR (e.g. May–July 2016 consultation on international development^2^). While steps have been taken to report foreign aid spending in line with international standards [42] and to post open data spreadsheets of basic project information, transparency on policy decision-making, resource allocations, grant review processes and outcome results could be drastically improved.

### IDRC

IDRC’s strategic framework and reports are made publicly available as part of an explicit acknowledgement that these documents are important tools for internal stakeholders to plan and assess their work. Further, the agency states that these documents are “*also an important tool for communicating with and providing accountability to stakeholders outside the* [IDRC]” ([18], p. 1-1). Historically, this Crown corporation has enjoyed an excellent reputation among international funders. IDRC continues to hold a broad set of development research priorities such as agriculture and the environment, economic growth, social policy, science and innovation, and global health. The latter commanded CA$16 M or 10% of IDRC’s total budget in 2012–2013 [43]. Unfortunately, the budget was reduced in the 2013 federal budget, and it has experienced several structural changes in recent years that may potentially have a negative effect on GHR. In 2014, IDRC launched a new Innovating for Maternal and Child Health in Africa programme that will fund implementation research teams and health policy research organisations on research related to MNCH [44].

Both our policy analysis and contributors to deliberative processes (including the 2013 dialogue and subsequent deliberations) indicated alignment between IDRC policy and practices and the equity-centred criteria used in this analysis. This was particularly true for the criteria of authentic partnering, inclusion, shared benefits and commitment to the future. One of IDRC’s recognised strengths lies in its commitment to “*devolve the responsibility for coordinating, managing, and administering research programs to Southern institutions whenever opportunity exists*” in order to build local capacity in research management ([18], p. 2-1). IDRC also declares its intention to support initiatives through the full-life cycle of programmes, with an estimated 2:1 ratio of funding for existing programmes compared to new concepts, and only a 35% portfolio involving Canadians [45]. IDRC should be applauded for championing this approach and should be encouraged to build on lessons learned in the implementation of its new 2015–2020 strategic plan [46]. On the other hand, IDRC does not appear to disclose results or impact using a results-based framework. Some data is available on its website and, although incomplete, some data on funded projects can be found on other open data sites. For example, information on funding recipients of the GHRI is posted on the National Institutes of Health World RePORT [47].

### CIHR

Canada’s largest health research funder, CIHR, continues to undergo transformations that were initiated in 2014. The CIHR 2010–2014 strategic plan set the lofty goal of ensuring Canada’s ongoing leadership in health research. GHR, particularly research that focuses on the social determinants of global health and global health processes, was even more concentrated within CIHR when, in 2009, the Social Science and Humanities Research Council (SSHRC) declared it would no longer fund social sciences-based health research [48]. CIHR has deemed topics related to GHR as priority areas for its competitions and provided funding contributions to joint initiatives such as the GHRI and GCC. CIHR is currently implementing reforms to its research-funding mechanisms. Three-quarters of funding will be directed towards the new ‘foundation scheme’ supporting established investigators and one-quarter towards a ‘project scheme’ supporting stand-alone research proposals by Canadian researchers more broadly [48, 49]. The reforms include the introduction of a new College of Reviewers in order to address issues with the grant review process. There may be opportunity to recommend reviewers with specific expertise in GHR. Unfortunately, the reforms to date have drawn vocal criticism from the research community. The urgency of criticisms from Canada’s scientific community [50] evoked an unprecedented response from Canada’s minister of health, Dr Jane Philpott, who called for an emergency meeting in CIHR to address the concerns and inform the Review of Federal Support for Fundamental Science [51]. These recent events present key opportunities for informing funding policies and practices that prioritise ethical and equitable engagement in GHR.

Unlike the United States National Institute of Health and United Kingdom’s Medical Research Council, there is no institute or division within CIHR dedicated to GHR, although the Institute of Population and Public Health emphasises health equity-oriented research [52]. While a report from DFATD indicated that a new CIHR global health strategy was developed in 2012–2013 [30], the only related publicly available document is an outdated Framework for International Relations and Cooperation from 2006 [53]. In this document, CIHR reported that “*operating grants with an international connection* [were] *overwhelmingly — about 90 per cent — with U.S. collaborators*” ([53], p. 5). Between 2005 and 2010, more so than other funders, CIHR’s goals were linked with driving Canadian economic growth through science and technology and protecting Canadians from emerging global threats versus improving population health, reducing health inequities and building capacity in LMICs. Encouragingly, following the end of the term of Dr Nancy Edwards as the Scientific Director of the Institute of Population and Public Health, her successor, Dr Steven Hoffman, will act as Scientific Lead for Global Health in addition to being the institute’s Scientific Director. This, as well the recent inclusion of CCGHR Principles for CIHR in training materials for the CIHR College of Reviewers^3^ are encouraging signs of the desire for investing in excellent and equitable GHR.

CIHR does not directly publicly disclose the portion of its CA$1.0 B annual budget contributed to GHR, though the numbers can be indirectly accessed through the Canadian Research Information System and they have also been featured as part of many past public presentations. A search of this system suggests that CIHR’s annual expenditures on GHR grants average CA$14 M, or 1–2% of its annual budget [54]. At a recent presentation, Dr Hoffman highlighted the growth in both absolute numbers and grant dollars among CIHR grant recipients between 2000 and 2015 with relevance to GHR (Dr Steven Hoffman, presentation to CCGHR Ontario Coalition Institute participants and facilitators, 2016). Despite the lack of a current, publicly available strategic plan for GHR, CIHR’s current strategic plan briefly mentions an interest in trainee success within GHR and a continued interest in global health issues under the research priority “*promoting a healthier future through preventative action*” ([55], p. 37). These are encouraging signs of a receptive funding environment at CIHR. One important step that could be taken by CIHR is toward making reports on GHR investments public, particularly as a demonstration of transparency and means for incentivising higher quality alignment of research proposals. In light of recent federal developments and the Review of Federal Support for Fundamental Science and open consultation on Canada’s international development policies, we are optimistic that CIHR’s next strategic plan will demonstrate a more explicit integration of global health and GHR across all of the institutes.

These would be welcome shifts, given the concerning language in the current CIHR strategic plan that listed many of the global health actions as predicated on the need for new funds [49]. Unlike IDRC, GCC and Global Affairs Canada, the current CIHR grants management policies do not disburse grant funds directly to LMIC research institutions; however, the policies do enable the transfer of funds from an eligible Canadian institution to a partner outside of Canada [56, 57]. That CIHR does not compensate investigator salaries [56] was perceived by participants at the 2013 CCGHR deliberative dialogue as a key barrier to enabling GHR, particularly for inhibiting the capacity of research institutions in LMICs to dedicate their time to an international partnership. Furthermore, participants believed that CIHR policies restrict the capacity to compensate the indirect costs incurred by non-Canadian partners in GHR. Participants argued that these restrictions were not realistic or equitable, particularly in the context of multi-country partnerships and in light of existing international standards that provide up to 20% or up to the full economic cost of projects. These concerns shed light on the disconnects between perceived and actual barriers, and present an opportunity for CIHR to consider ways in which they might clarify their policies and incentivise universities to consider the role academic institutions can play in enabling equity in GHR partnerships that involve LMICs. For these reasons, we found CIHR policies to show emerging alignment with the equity-centred criteria outlined in Table 1.

### GHRI

The GHRI, created in 2001, was one of the first attempts at a coordinated approach to GHR funding between Health Canada, CIDA, IDRC, the Public Health Agency of Canada and CIHR. This initiative, and its flagship CA$25 M Teasdale-Corti programme, led to new multi-year research partnerships between LMIC- and Canada-based researchers with a focus on building applied health research capacity for researchers in poor countries. Egalitarian partnerships and a concerted effort to build local capacity were central to the design of this new funding model [23, 58]. However, structural differences in the amount of funding support provided and grants management policies between the founding partners of GHRI created certain complications in GHRI’s operations, and potentially to its absorption into IDRC. Despite these challenges, however, GHRI demonstrated to funders and researchers alike that there was potential for unconventional approaches to stimulating GHR in Canada and abroad.

### GCC

In 2010, the Canadian government created a CA$225 M Development Innovation Fund to be disbursed through the new GCC to spur innovation by global health innovators in LMICs and Canada [45]. The entity is guided by IDRC, CIHR and Global Affairs Canada (previously DFATD) [59]. GCC’s approach, modelled after the BMGF grand challenges approach, uses funding mechanisms that contrast sharply with those of its founding government bodies. Being an independent entity affords it the ability to undertake aggressive and unconventional approaches to funding innovation [60, 61]. In general, it promotes biomedical and technology-based research, product development and rapid scale-up. GCC targets health gaps in LMICs such as point-of-care diagnostics, improving birth outcomes, brain development and mental health. Between 2012 and 2014, GCC was, on average, the largest source of GHR funding in Canada, reaching a peak of CA$54 M disbursed in 2013 [62].

It was difficult to find clear evidence of alignment between GCC policies and practices and the equity-centred criteria used in this analysis. The short-term project focus of GCC grant competitions, combined with a focus on technology and innovation, did not align with the criteria for responsiveness to causes of inequities and raised questions about the possibility of enabling shared benefits. GCC’s strengths lie in its focus on saving lives in LMICs, alignment with the US-based BMGF and United States Agency for International Development approaches, and its ability to engage the public, policymakers and research institutions in Canada and around the world. Importantly, GCC also has the capacity to disburse funds directly to LMIC researchers following an institutional assessment of financial management capacity [61]. GCC does not have a publicly available strategic plan, which inhibits potential partners from clearly understanding its long-term vision, mission and strategies. It has recently posted portions of a Results-Based Management Accountability Framework [62], but it neither provides sufficient information on how data are generated nor shows targets for such indicators. GCC has showcased some examples of project failures in the 2013–2014 Annual Report, a positive step towards sharing important learnings – although it is likely that valuable lessons could be learned from the experiences of failures that did not fit the profile of so-called ‘fast failures’. The dynamic and unconventional GCC has been a welcome funding boost for biomedical science and technology researchers in GHR. GCC has the opportunity to truly set a new benchmark for the level of transparency among funding agencies in Canada and abroad.

### A disjointed strategy for GHR

After the launch of the 2010 Muskoka MNCH Initiative, the Canadian Academy of Health Sciences argued that, unlike Norway, the United States and the United Kingdom, Canada did not have a unified vision for global health. Through a wide-reaching consultative process, the Academy proposed that a strategic role for Canada in global health should be based on Canadians’ strong value for universal healthcare, a vibrant philanthropic sector and strong commitment to MNCH as key strengths [5]. It was also noted, however, that poor coordination among Canadian global health actors, limited application of our understanding of social determinants of health to policies and actions, and resource constraints within government, private and civil society sectors would be barriers to optimal coordination.

Participants involved in deliberative dialogues as part of the 2013–2014 CCGHR Gathering Perspectives Study raised several concerns for Canada’s approach to GHR. In particular, there was concern for how the current division of roles among Canadian funding bodies seemed to fuel an uncoordinated GHR strategy and contradictory granting policies. Additionally, the community raised concerns of the levels of tied aid^4^ and research, which may not respond to local needs and risks a neo-colonial development approach, and cautioned that academic interests should not usurp benefits from local communities. They called for a unified vision for GHR, for greater collaboration within Canada and with its partners in other countries, and for a deeper commitment to equity-centred GHR [6].

### Best practices from international funders

There are a number of characteristics and best practices for funding GHR that were identified from the literature, revealing a sample of government and philanthropic agencies based in Australia, Denmark, Norway, Sweden, the European Union, the United Kingdom, and the United States.^5^ One characteristic of the Canadian international development structure that differs from Australia, Norway, Sweden, the United Kingdom and the United States is the separation of the development programming agency or branch (Global Affairs Canada, formerly DFATD and CIDA) and its aid research arm, the IDRC, which may conceivably impede coordination or gains from synergies. Most comparator countries invest proportionately more in GHR than Canada. Several Scandinavian countries, the Netherlands, and the United Kingdom exceed the United Nations target of 0.7% ODA/GNI for aid spending [36].

The United States is traditionally the single largest DAH donor nation, although US bilateral assistance fell 7.2% from 2011 to 2012 and then a further 3.4% from 2012 to 2013, due to budget sequestration [14]. The United States Agency for International Development (USAID) budget alone includes US$5 B for health sector activities [20]. It places a high value on strategies that build LMIC ownership and invests in science, technology and other research activities, including health systems research. In recent years, the agency has placed greater emphasis on evidence-based strategies and promoted dialogue about learning from failure. Similar to DFATD, the agency supported MNCH as a priority. In contrast, however, USAID supported family planning and sexual reproductive health initiatives as well as a programme designed to reach marginalised groups such as lesbian, gay, bisexual and transgender persons and men who have sex with men [20, 63]. However, this support is subject to political shifts and may now be under revision by the current United States government. The long-standing Fogarty International Centre has led the coordination of all NIH-funded GHR and capacity-building activities. It has a clear strategic plan for GHR, which emphasises the importance of implementation and social science research in addition to biomedical, scientific and technological approaches. Further, it encourages capacity building with LMIC institutions across its programmes and, importantly, releases direct funding to LMIC institutions [64].

The last decade has also seen a dramatic rise in the strategic and financial influence of private philanthropic organisations in the United States. The Clinton Foundation’s global health budget of US$134 M in 2012 was focused on increasing access to treatment for HIV/AIDS, malaria and diarrheal diseases, along with lowering costs for essential medicines and supporting health systems infrastructure in LMICs [65]. The BMGF, founded in 2000, has quickly become the largest philanthropic foundation globally and funder of global health activities, including GHR (up to US$1.8B for global health in 2012) [66]. Both organisations leverage the advantages of being a private entity, including the ability to invest in high-risk or long-term initiatives as well moving freely between public and private partners. The BMGF openly states their criteria for investment, and actively discloses and shares lessons learned from major failures, challenges and lost investments [66]. The BMGF also administers an annual independent grantee survey to learn from partners. Additionally, the foundation encourages grantees to publish any and all findings from funded activities [66]. One weakness that the foundation has identified is that the publicly available information on grantees is not in a format that is easily analysable.

In 2012, the European Commission launched a new €80 B (CA$115 B) Horizon 2020 research funding scheme. Although GHR is not central to the programme, one of its strategic directions is framed broadly as “*tackling global societal challenges*” ([67], p. 4). Horizon 2020 has adopted a novel policy that considers all LMIC researchers as “*automatically eligible non-EC applicants*” ([67], p. 4) to compete for funding. This may indirectly spur research and innovation in LMICs where researchers are considered to be at a disadvantage due to the absence of a critical mass of researchers and local research-funding infrastructure [68]. The United Kingdom’s primary GHR funders are the Department for Foreign Affairs and International Development, the Medical Research Council and the Wellcome Trust. The Department for Foreign Affairs and International Development has an integrated Research and Evidence division with a substantial budget of £405 M per year [19, 69]. In this budget, 10% of its DAH, or £50 M per year, is allocated to research. The departments’ rationale for a recent research budget increase was to “*make sure that research is at the heart of our work to influence the development community, we want to use it to better shape our own policy and programmes*” ([19], p. 13). The Medical Research Council also has a strategic direction entirely dedicated to global health, with the objective of supporting “*global health research that addresses the inequalities in health which arise particularly in developing countries*” ([70], p. 3).

Denmark, Sweden and Norway are proportionally the highest contributing Development Assistance Committee countries, achieving a 0.85%, 1.02% and 1.07% ODA/GNI ratio, respectively. These ratios are far above those of other Development Assistance Committee countries and above the United Nations target of 0.7% [36]. Even though they both have much smaller populations, Sweden and Norway each made larger total aid spending (over CA$5 B) than Canada did in 2013. Norway’s Global Health in Foreign Aid and Development Policy sets an example for others by articulating the values, goals, priorities, rationale and approaches the country employs in administering a unified national global health strategy [17]. Central to its approach is the concept of ‘knowledge-based policy’, which requires the systematic use of research-based knowledge to evaluate measures and continuous monitoring using information systems for health data. The policy also recognises that “*innovation poses particular challenges for knowledge-based policy formulation*” ([17], p. 40) due to the lack of evidence for new, high-risk initiatives. The Swedish International Development Agency strategy emphasises the value of the reciprocal benefits of pairing a research agenda with implementation of development programmes. It uniquely stresses the importance of conducting research on an equal footing with LMIC partners, stating that “*research support should be designed in such a way that it helps prevent the development of a superior and an inferior status in this relationship*” ([70], p. 19; [71]). The Danish International Development Agency is the only funder to cite the limited public investment in research by developing countries (0.3% GDP on average) as a rationale for continued research-focused aid. As such, the Danish International Development Agency’s efforts focus on south-driven research [72].

Finally, the Australian Agency for International Development is focused on saving lives in low-resource countries in the Asia-Pacific region, and has committed to investing over AU$100 M over 5 years in its medical research strategy [73]. This agency’s strategic plan details its criteria and prioritisation process for funding medical research projects. Also of note is the increasing support by emerging donor (Brazil, Russia, India and China) countries in DAH, including the transfer of technology and private investments to low-income countries over the past few decades. However, “*little is known about the magnitude and scope of DAH provided by some of the emerging development assistance partners*” ([14], p. 60).

### Implications for Canadian involvement in GHR

This analysis revealed a complex and dynamic GHR funding landscape in Canada. Recent funding developments include renewed opportunities for GHR, including the 2017 SSHRC announcement that health-focused social science research would again be eligible for funding [74]. Given that both Global Affairs Canada and CIHR (and possibly other agencies) are undergoing review in 2016 and 2017, there may be a time-limited opportunity to enact changes, or even redesign the GHR funding system, necessary to support a vibrant GHR community in Canada. A meaningful redesign that advances equity as the central goal of GHR can only be achieved with caution, reflection and dialogue on the opportunities for nurturing promising GHR funding practices among funders, universities and grant seekers. Based on our analysis and deliberative processes, we believe there are important opportunities for key actors to contribute to elevating equity-centred GHR funding policy and practice (Table 3).

**Table 3:** Recommendations for funding policies and practices

Target audiences	Recommendations
National policy bodies (e.g. elected government, governmental committees) National GHR networks	Develop a national strategic plan for GHR Set benchmarks for dedicated research supports in Canadian investments in global health Promote research on research, including promising practices in GHR
Funding agencies (e.g. CIHR, GCC, IDRC)	Model transparency in GHR funding Create consistent GHR-friendly funding structures and policies Invest in communications about funding policies in ways that encourage equity-centred grant seeking and administration at the university level Open funding competitions to LMIC researchers
People involved in teaching, supporting, using, doing or funding GHR	Explicitly acknowledge a foundational commitment to equity in the health and well-being of populations, communities and individuals (e.g. guided by the CCGHR Principles for GHR)

*CCGHR* Canadian Coalition for Global Health Research, *CIHR* Canadian Institutes of Health Research, *GCC* Grand Challenges Canada, *GHR* global health research, *IDRC* International Development Research Centre, *LMIC* low- and middle-income countries

Universities have an important role to play in creating enabling environments for equitable management of GHR grants and for promoting more equity-centred GHR. Although participants in the CCGHR studies directed their concerns at funding agencies, some of the barriers they described were, in actuality, rooted in the policies of their institution’s grants administration. Universities can, for example, examine their internal policies and practices related to the administration of grants. In addition, universities can encourage flag equity as a consideration in pre-submission peer review. This could involve considering how equity is reflected in different elements of grant proposals, including budgets that enable capacity building and compensation for the contributions of partners outside of Canada. Tenure review processes that create a means for assessing equity in GHR are another important policy arena where universities can promote equity-centred GHR, including principles such as those outlined in the CCGHR Principles for GHR [4]. Finally, universities and researchers both carry a responsibility for demonstrating excellence and enhancing the visibility of GHR in open funding competitions. This means making an explicit effort to identify grant applications as relevant to GHR by using the terms ‘global health’ or ‘global health research’ in abstracts, keywords and the body of grant applications. These steps could both improve the capacity of funding agencies to consistently report investments in GHR and improve the overall competitiveness of GHR.

Greater harmonisation between Canada’s research and development funders on GHR priorities and activities could be enhanced through a joint national strategic plan, setting benchmarks for GHR funding, and with a long-term commitment to the strategy. A coordinated GHR strategy, if not more broadly for global health and development, would need to include Global Affairs Canada, CIHR, IDRC, SSHRC and GCC. Such a strategy would also benefit from contributions from bodies like the Canadian Academy of Health Sciences, the Canadian Society for International Health, the CCGHR, the Canadian Council of International Cooperation, the Canadian Network for Maternal, Newborn and Child Health, and major Canadian philanthropic foundations and academic institutions. A unified GHR strategy, and the strategies of each of its contributors, should have an integrated evaluation framework that incorporates qualitative research methods and policy analysis. Additionally, bibliometrics and spending data is needed to measure achievements and discern best practices such as the Health Economics Research Group payback model recommended by CCGHR [75].

Given the fragmentation and partisan influence behind the funding shifts that followed the 2008 recession, earlier calls for attention to the absence of a national strategy for global health [5], and continued transformations in the Canadian funding landscape, this is an opportune time to re-evaluate Canada’s strategic position and contributions to GHR. Participants in both the 2013 CCGHR deliberative dialogue and the deliberative processes used to validate this analysis agreed. This strategy could enable Canada to model the advancement of transparency in strategic planning, decision-making and disbursements. Central aims of such a strategy could include greater transparency with partners and stakeholders in LMICs through engagement, better alignment developing country partner priorities and adherence to the principles of aid effectiveness established in the 2008 Accra Agenda for Action [76], namely ownership, inclusive partnerships, delivering results and capacity development [77]. LMIC researchers who partner with Canadian institutions or researchers to do GHR face “*varying stakeholder expectations, unaligned grant cycles, and highly variable reporting requirements*” ([77], p. 1). Canadian funders must strive for a GHR-friendly grants management policy framework that places LMIC researchers on an equal footing. This may also necessitate a review of Global Affairs Canada’s engagement with the mining industry in corporate social responsibility programmes [34].

Canadian funders should also not limit the scope of interventions and research investigations related to such topics based on social, religious or political ideologies (i.e. sexual and reproductive health and rights), which are central to equity-oriented GHR. It is important that GHR investments remain balanced between research on innovation of biomedical sciences and technology while continuing to strengthen the fundamentals of health systems and health equity. This kind of balance will also require broad inputs from health policy research and social, economic and environmental sciences. Canadian funders should promote open sharing of knowledge including failures, an essential aspect of learning. Research grantees, aid recipients and evaluators alike should be encouraged to disseminate knowledge and share lessons learned from both successes and failures with the broader Canadian and international health community. These structural changes may help reduce some of the unintended consequences of foreign aid and GHR.

Lastly, Canada would be wise to rectify the reputational and operational damage stemming from the 11% foreign aid budget cut in 2012–2013, and ensure that the new MNCH commitment of funds (CA$3.5 B between 2015 and 2020, or CA$700 M per year) is truly new money relative to 2011–2012 levels. Global Affairs Canada should also reconsider whether the amalgamation of development programming with trade and foreign affairs branches presents long-term risks to its effectiveness and ethics in aid delivery, and take this opportunity to imagine an architecture for consolidating all global health and development (including research) funding under one independent agency, free of political influence.

### Limitations

We acknowledge certain limitations in conducting this policy analysis. First, the policy analysis was conducted primarily using non-peer reviewed government and non-government publications available in the public domain. The content of documents varied by funding body, making it difficult at times to compare information. This process required an assumption that the majority of information contained in the documents is accurate. Second, the lack of information on new GHR funders from Brazil, Russia, India, China and the private sector limited the analysis to Western governments and major philanthropic funders.

## Conclusion

With a review of strategic planning undertaken in all main Canadian funders in 2016, there is an important window of opportunity for the GHR community, in Canada and abroad, to influence policy towards a funding environment that is reflective of foundational principles for equity-centred GHR. This analysis of the current GHR funding landscape and promising practices internationally has informed the development of a core set of recommendations by CCGHR (Table 3). A national strategic plan for GHR would be strengthened by the inclusion of benchmarks or targets for Canadian GHR funding and the promotion of research on research. Canadian funding agencies can enhance their contributions to equitable GHR funding by modelling transparency, clarifying and encouraging equity-centred funding policies, and opening funding competitions to LMIC researchers. Universities and researchers can consider their own roles in placing equity at the centre of their GHR practices and policies, explicitly acknowledging a foundational commitment to equity in the health and well-being of populations and communities. Continued demonstration for the importance and value of long-term, stable funding (looking to exemplars of international comparators) falls to the GHR community and is a critical contribution to the redesign of global health funding systems. Together, the multiple players involved in shaping the funding landscape in Canada can realise these recommendations and doing so will advance Canada’s collective contribution to improving health equity globally.

## Abbreviations

BMGF: Bill and Melinda Gates Foundation
CCGHR: Canadian Coalition for Global Health Research
CIDA: Canadian International Development Agency
CIHR: Canadian Institutes of Health Research
DAH: development assistance for health
DFATD: Department of Foreign Affairs, Trade and Development
GCC: Grand Challenges Canada
GHR: global health research
GHRI: Global Health Research Initiative
GNI: gross national income
IDRC: International Development Research Centre
LMIC: low- and middle-income country
MNCH: maternal neonatal and child health
ODA: official development assistance
OECD: Organisation for Economic Cooperation and Development
SSHRC: Social Sciences and Humanities Research Council
USAID: United Sates Agency for International Development.

## Footnotes

1 This Gathering Perspectives (GPS1) project was funded by the Global Health Research Initiative (GHRI).

2 For more information, visit: http://international.gc.ca/world-monde/issues_development-enjeux_developpement/priorities-priorites/what_we_heard-que_nous_entendu.aspx?lang=eng

3 These modules include the global health research and unintentional bias, and are openly accessible (toggle the tab for "Peer Review" to access a complete listing): http://www.cihr-irsc.gc.ca/e/47021.html

4 Tied aid is the practice of tying official development assistance to the trade interests of the donor country by, for example, setting a condition of spending aid on export goods from the donor country.

5 The research team declares that this analysis is limited by the availability and transparency of information in documents available in English from donors predominantly based in the West.
